# Knowledge of Herpes Zoster and Attitudes Toward Vaccination: A Questionnaire-Based Study in Outpatient and Online Settings

**DOI:** 10.3390/vaccines14090834

**Published:** 2026-09-21

**Authors:** Michał Oleszko, Artur Mazur, Lech Zaręba, Nikola Król, Aleksandra Łoś, Hanna Czajka

**Affiliations:** 1 College of Medical Sciences, University of Rzeszów, 35-315 Rzeszów, Polandhanna.czajka@onet.pl (H.C.); 2 Institute of Computer Science, University of Rzeszów, 35-315 Rzeszów, Poland

**Keywords:** herpes zoster, vaccination, vaccine hesitancy, knowledge, attitudes, CART analysis, Poland, primary care, cross-sectional study

## Abstract

Background/Objectives: Herpes zoster (HZ) vaccination rates remain low globally despite the availability of effective recombinant vaccines. This study aimed to assess knowledge of HZ and HZ vaccination, willingness to vaccinate, and factors associated with vaccination willingness among at-risk populations in both outpatient clinic and online survey settings in Poland. Methods: A questionnaire-based cross-sectional study was conducted between May and September 2025 in outpatient primary care clinics operated by Medical Center Medyk (Podkarpackie Voivodeship, southeastern Poland) and via online social media platforms. Participants included individuals aged ≥50 years with a history of HZ and individuals aged ≥18 years with at least one recognised HZ risk factor. Multivariable logistic regression and classification and regression tree (CART) analysis were employed to identify predictors of vaccination willingness. Results: Valid responses were obtained from 212 onsite and 241 online participants. A prior history of HZ was significantly associated with correct responses to several knowledge items concerning HZ and HZ vaccination in both settings. Over 40% of initially hesitant or unwilling onsite participants reported a positive change in vaccination intention following medical consultation. CART models demonstrated good discriminative performance (AUC: online 0.841, onsite 0.861). The primary difference between settings was the source of information: online participants relied more heavily on the internet, whereas onsite participants more frequently cited healthcare professionals. Conclusions: Personal experience with HZ and engagement with healthcare professionals significantly influence vaccination willingness. Targeted educational interventions delivered through appropriate communication channels, with emphasis on physician engagement, may help improve vaccination willingness among at-risk populations.

## 1. Introduction

Herpes zoster (HZ, shingles) is an infectious disease caused by the reactivation of the latent varicella-zoster virus (VZV) [1], which remains dormant in neural tissue [2,3,4] following primary infection with varicella (chickenpox), typically acquired during childhood [5,6,7,8,9,10]. Reactivation is generally believed to result from a decline in VZV-specific immune surveillance [11,12]. The virus may disseminate systemically, leading to complications such as postherpetic neuralgia, vasculitis, retinitis, and enteritis. The risk of stroke increases following an episode of HZ, particularly in patients with herpes zoster ophthalmicus (HZO) and in those who are immunocompromised [13]. Reactivation of VZV may result in cranial neuropathies, focal muscle weakness, encephalitis, or partial facial paralysis [14,15]. HZ oticus results from the spread of VZV to the vestibulocochlear nerve and may present with symptoms such as vertigo and hearing loss [16]. HZO occurs in up to 20% of patients with HZ and involves the ophthalmic division of the trigeminal nerve, potentially leading to ocular complications and visual impairment [17,18,19,20,21].

Due to its associated complications, HZ represents a significant public health concern, particularly among older adults. However, HZ can be prevented through vaccination. The first licensed vaccine, the live attenuated zoster vaccine (ZVL; Zostavax), was approved in 2006 for adults aged ≥60 years [22,23] and subsequently approved for adults aged 50–59 years in 2011 [22,24,25]. Recommendations issued by the US Advisory Committee on Immunization Practices (ACIP) were not expanded, however, due to insufficient evidence of vaccine effectiveness in the 50–59-year age group [26,27].

Due to safety concerns in immunocompromised individuals—including patients with immunodeficiency disorders, those receiving immunosuppressive therapy, and pregnant women—ZVL is contraindicated in these populations, as it contains live attenuated virus particles [25,28,29,30].

In 2017, the recombinant zoster vaccine (RZV; Shingrix) was approved by the US Food and Drug Administration (FDA). RZV is non-live and recombinant, containing a modified VZV glycoprotein E (gE) with the AS01B adjuvant system. ACIP recommendations were subsequently expanded to include individuals aged ≥50 years. In 2021, RZV was approved for use in immunocompromised individuals, and ACIP recommended a two-dose regimen for immunocompromised persons aged ≥18 years [31,32,33,34,35,36].

Despite well-established benefits of vaccination, some patients remain unwilling to be vaccinated [24,37]. Reasons include lack of knowledge, doubts about vaccine effectiveness, financial constraints, and limited contact with healthcare providers [37,38,39,40,41,42].

Due to these factors, HZ vaccination rates vary globally and remain low in many countries [37]. In the United States, only 24.1% of adults aged ≥50 years had received any HZ vaccine in 2018 [43], rising to 34.4% of all eligible patients in 2022 [44]. In England, cumulative HZ vaccination coverage among individuals aged 71–80 years ranged from 43.6% (71-year-olds) to 83.4% (79-year-olds) as of late September 2023 [45]. In Italy, particularly in island regions, vaccination coverage is extremely low, estimated at 2.9% among eligible individuals [46]. In Israel, vaccination coverage among patients with rheumatic diseases was only 3.6% [47].

The primary aim of this study was to assess whether a history of HZ, symptom severity and duration, comorbidities, lesion localization, and treatment modalities are associated with knowledge of HZ and HZ vaccination, as well as willingness to vaccinate, in both outpatient and online survey settings. Secondary objectives were to compare demographic and clinical characteristics between online and onsite groups and to evaluate whether medical consultation among vaccine-hesitant individuals was associated with changes in vaccination decisions.

## 2. Materials and Methods

### 2.1. Study Design and Population

We conducted a questionnaire-based cross-sectional study (Supplementary Material Table S1) in outpatient primary care clinics and via online recruitment. The study was conducted concurrently in onsite and online settings between May and September 2025. In outpatient settings, participants were randomly selected from the primary care network operated by Medical Center Medyk in the Podkarpackie Voivodeship, southeastern Poland—one of the largest healthcare providers in the region, operating 20 primary care facilities across the voivodeship.

Eligible participants included individuals aged ≥50 years with a history of HZ, regardless of comorbidities, and individuals aged ≥18 years belonging to HZ risk groups (defined as having at least one of the following: congenital or acquired immunodeficiency, including iatrogenic immunosuppression and malignancies such as leukemia, lymphoma, or multiple myeloma; solid organ or hematopoietic stem cell transplantation; hypertension; chronic heart, liver, lung, or kidney disease; autoimmune diseases; diabetes mellitus; or depression). Control groups comprised individuals without a history of HZ meeting the same inclusion criteria. For the purposes of this study, the listed conditions were used as eligibility criteria because they are recognized as conditions associated with an increased risk of HZ in the Polish multidisciplinary expert recommendations on HZ vaccination [48] and in the Herpes Zoster Comorbidities Guide by GSK [49]. In addition to the well-established HZ risk factors, depression and hypertension are also recognized in the cited sources as comorbid conditions associated with increased HZ risk and were therefore included among the study eligibility criteria. The cited documents were used to characterize HZ risk rather than to define formal vaccination indications or recommendations, which may vary according to the applicable clinical guidelines.

The questionnaire was developed specifically for the purposes of this study and was not adapted from an existing validated instrument. The same questionnaire items, response options, wording, and order of presentation were used in the online and onsite settings. The selection of domains relating to HZ knowledge, vaccine awareness, vaccination attitudes, and barriers was informed by previous questionnaire-based studies [50,51,52,53,54,55]. Questions concerning HZ risk factors and populations at increased risk were also consistent with current clinical recommendations [48]. The draft questionnaire was reviewed by senior members of the study team with substantial clinical and research experience in HZ and vaccination to assess the relevance, clarity, and completeness of the items. It was subsequently pretested in a small group of patients to assess the clarity and comprehensibility of the items and the practical feasibility of administration. Feedback was used to refine the wording where necessary. No composite knowledge score was constructed, and individual knowledge items were analysed separately. No formal psychometric analyses were performed at this stage. For each knowledge item, the correct response was specified before data analysis on the basis of established clinical knowledge.

For the online survey, the questionnaire was distributed via social media groups and internet forums (access restricted by terms of service and requiring administrator approval) and was available only to individuals meeting the eligibility criteria. Prior consent was obtained from group administrators before posting the survey. The risk of receiving duplicate responses was minimized by allowing only one completed survey per IP address. A set of logically related validation questions was included to ensure response consistency. Respondents who provided contradictory answers to these items were excluded from analysis.

In the onsite group, a physician-led consultation was conducted after completion of the questionnaire and lasted approximately 15 min. It followed routine clinical practice and focused on HZ, its potential complications, and available preventive measures, including vaccination. Vaccine efficacy, safety, indications, reimbursement, and practical aspects of vaccination were systematically discussed during the consultation, although no standardized script was used. Physicians were aware of participants’ baseline questionnaire responses. The post-consultation assessment was completed on the same day.

**Figure 1:**
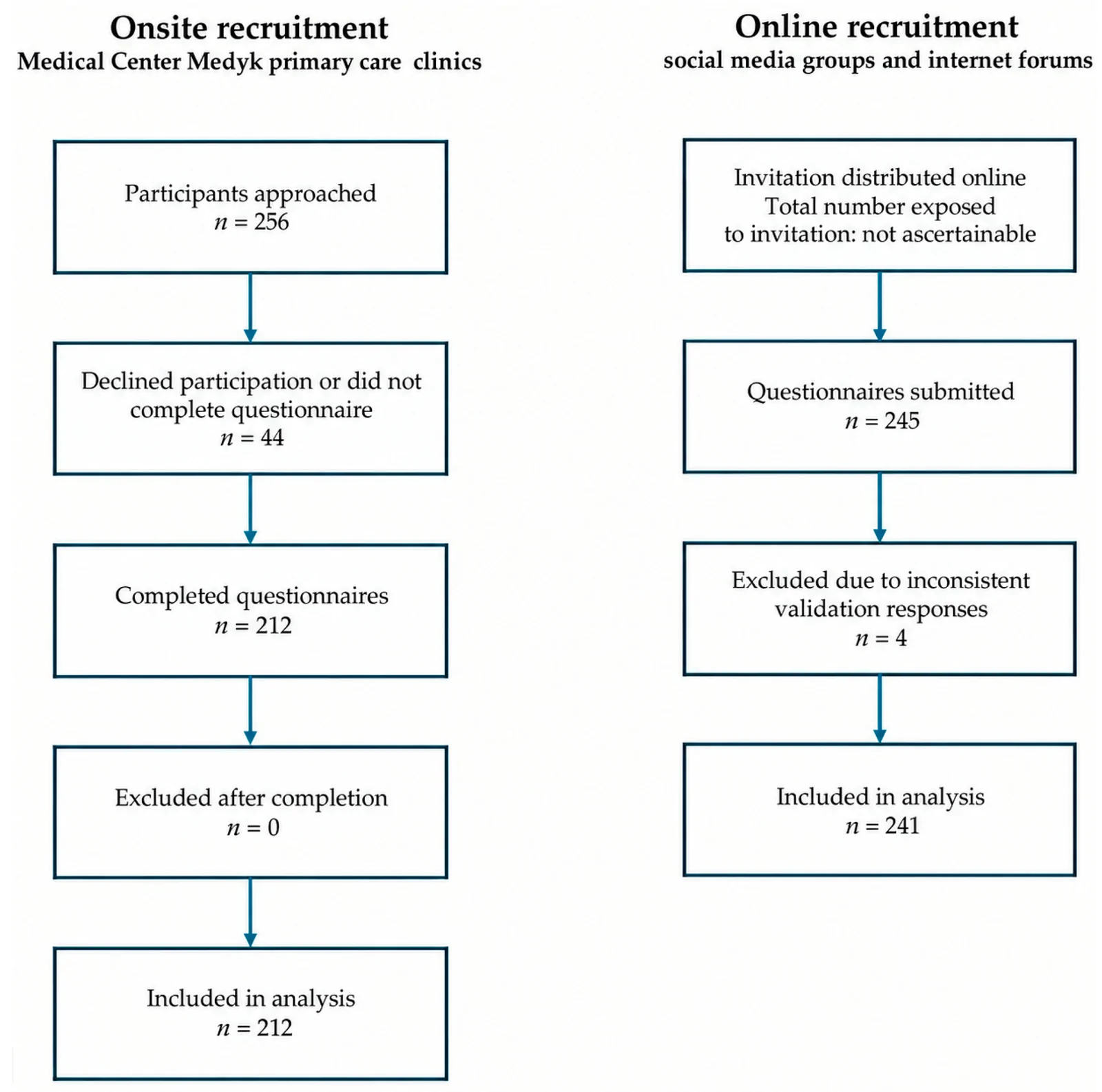
Participant flow for the onsite and online recruitment settings.

Onsite participants were randomly selected using a computer-generated random sampling procedure. If a selected individual declined participation or could not be contacted, another participant was randomly selected using the same procedure. Recruitment in both settings aimed to obtain broadly comparable numbers of participants with and without previous HZ, although the final distribution was more balanced in the onsite group than in the online group. Participant flow for the onsite and online recruitment settings is presented in Figure 1.

In addition to clinical variables, sex, age, and place of residence were collected. Urban areas were defined as cities with >100,000 inhabitants, suburban areas as smaller towns with municipal status, and rural areas as non-urbanized localities without municipal rights.

### 2.2. Statistical Analysis

We used STATISTICA Tibco 13.3 software (StatSoft Inc., Tulsa, OK, USA) and R program (version 4.3.1) to analyze the data. Categorical data were evaluated using either the Chi2 test or Fisher’s exact test, depending on applicability, and results were expressed as counts (*n*) alongside their corresponding percentages (%).

Model selection for logistic regression was based on the Akaike Information Criterion (AIC). Variables were selected for inclusion in the multivariable analysis based on their statistically significant association with the dependent variable, together with additional variables selected on the basis of clinical relevance. These included age, sex, place of residence, and chronic diseases. Subsequently, based on the statistical analyses and model-fit measures, the set of variables that provided statistically significant results and the best model-fit parameters, while avoiding multicollinearity as assessed using the variance inflation factor (VIF), was retained in the multivariable models. Models including only disease history were compared with fully adjusted models (sex, age, and place of residence) and with models selected via best-subset selection. In some cases, adding age or sex yielded marginally lower AIC values; however, this improvement was not consistent across outcomes. Net Reclassification Improvement (NRI) was used to assess whether adding age, sex, and place of residence improved classification performance; NRI did not indicate significant improvement over simpler models in any case. Therefore, odds ratios from univariable logistic regression models including only disease history are reported.

The candidate predictors considered for the CART analysis included sex, age, place of residence, comorbidities, e.g., hypertension, diabetes mellitus, autoimmune thyroiditis, as well as the categories “none of the listed conditions” and “any comorbidity.” Other candidate predictors included a history of HZ and responses to questionnaire items assessing whether HZ is contagious, which disease is caused by the same virus as HZ, whether individuals aged ≥50 years are at a higher risk of HZ, whether individuals with chronic diseases are at a higher risk, whether a HZ vaccine exists, the source of information about the vaccine, and the perceived role of HZ vaccination. Candidate predictors were selected based on clinical judgment. Missing data occurred only for the dependent variable, and participants with missing values for this variable were excluded from the analysis.

For the CART analysis, the Gini index was used as the splitting criterion, and the minimum node size was set at 5 observations. Cost-complexity pruning was applied using a complexity parameter (cp), with the optimal tree selected based on the lowest 10-fold cross-validated error. Class weighting was applied based on the percentage distribution of responses between the outcome classes. Model performance was additionally evaluated using bootstrap-based internal validation with 200 samples. The mean bootstrap optimism correction for accuracy was 0.05 in the online group and 0.08 in the onsite group. The AUC values were obtained from models generated using the bootstrap-based bagging procedure. Analyses were performed in R (v4.5.1) using the rpart (v4.1.27), rpart.plot (v3.1.4), caret (v7.0-1), and ipred (v0.9-15) packages.

### 2.3. Ethics

Institutional Review Board Statement: The study was approved by the Ethics Committee of University of Rzeszow (protocol code 2025/04/29/; date of approval 4 April 2025) and all consents required by the Committee were obtained.

## 3. Results

### 3.1. Baseline Characteristics

In the onsite group, a total of 256 participants were approached, of whom 212 responded and completed the questionnaire; no responses were excluded, resulting in 212 participants included in the final analysis. For the online group, the number of participants approached could not be determined because recruitment was conducted through social media groups and internet forums. A total of 245 participants responded, of whom four were excluded due to inconsistent answers in the validation items, resulting in 241 participants included in the final analysis. Baseline and clinical characteristics of both groups are presented in Supplementary Table S1.

### 3.2. Knowledge of Herpes Zoster and Vaccination

A prior history of HZ was significantly associated with correct responses to several individual questions concerning HZ and HZ vaccination, as assessed by questions 15–19 and 21. The ratio of correct to incorrect responses, together with corresponding test results for questions showing statistical significance in at least one setting, are presented in Table 1. “I do not know” responses were treated as missing for the purposes of statistical analysis and were not included in comparisons of correct versus incorrect responses.

Test statistics and *p*-values for individual questions concerning HZ severity, symptom duration, and complications in the online and onsite groups are presented in Table 2. Only questions showing statistical significance in at least one setting are reported. Statistically significant differences in responses to selected HZ-related knowledge questions were observed for complications only among clinic-based participants. Lesion localization was not significantly associated with knowledge of HZ or HZ vaccination. No significant associations were observed for disease recurrence or use of immunosuppressive therapy.

Table 3 presents the results of bivariable analyses of individual chronic diseases and responses to selected knowledge questions. Rare conditions represented by very small numbers of participants were treated descriptively and were not included in inferential analyses.

### 3.3. Changes in Vaccination Willingness Following Medical Consultation

Consultations were conducted only in onsite settings. Changes in willingness to vaccinate against HZ after medical consultation are shown in Table 4. Participants who did not change their opinion answered the same to question 23 (‘If you have not been vaccinated, do you plan to receive the HZ vaccine?’) before and after consultation. Positive changers answered ‘No’ before and ‘I don’t know’ or ‘Yes’ after consultation, or ‘I don’t know’ before and ‘Yes’ after. Negative changers answered ‘Yes’ before and ‘I don’t know’ or ‘No’ after, or ‘I don’t know’ before and ‘No’ after. Statistically significant associations between participant characteristics and changes in opinion are presented in Table 5.

### 3.4. Differences Between Online and Clinic-Based Populations

For most knowledge-related questions, responses did not differ significantly between the online and clinic-based groups, with the exception of the source of information about the existence of HZ vaccination (Table 6). Similarly, no significant differences were observed in reasons for accepting or refusing vaccination or in future vaccination intentions.

### 3.5. Reasons for Vaccine Hesitancy and Acceptance

The most frequently reported reasons for not being vaccinated are shown in Table 7. The most commonly cited were: lack of awareness that a vaccine exists (35.15% onsite, 32.24% online), concern about vaccine side effects (24.73% onsite, 26.23% online), and not receiving information from a doctor or medical staff (21.43% onsite, 20.77% online). The most frequently reported reasons for deciding to vaccinate are shown in Table 8.

### 3.6. CART Classification Models

**Figure 2:**
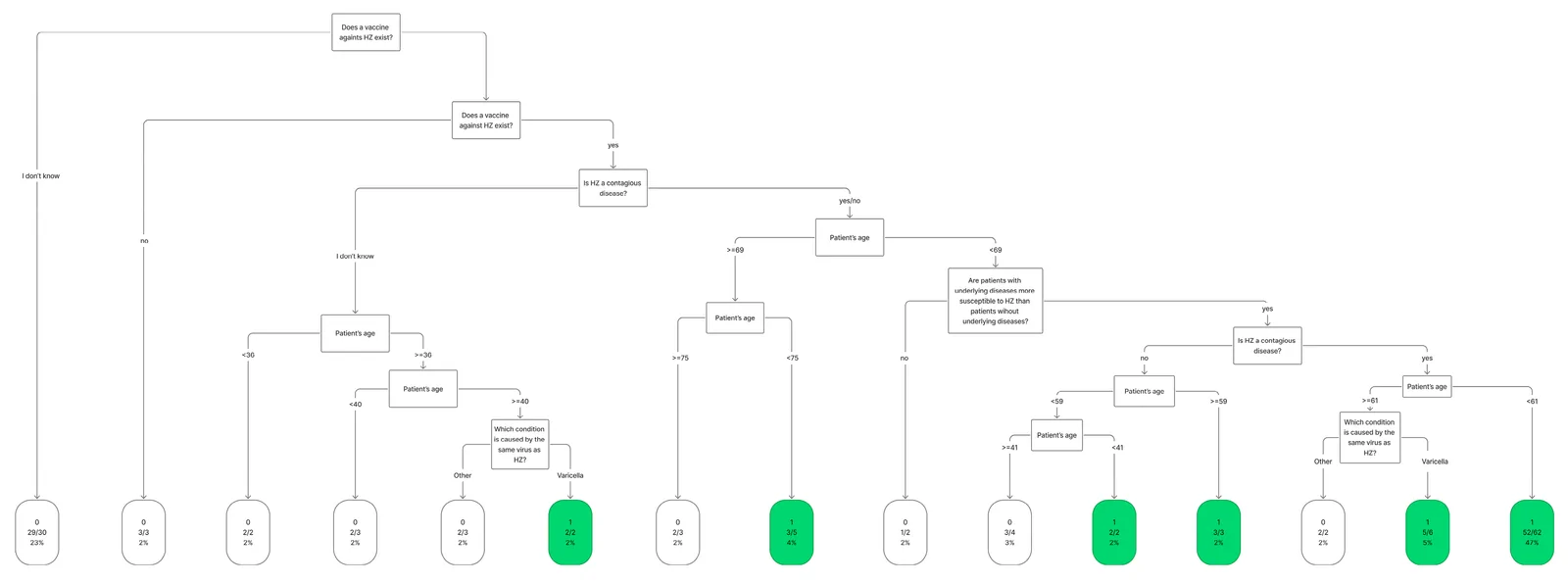
CART model predicting willingness to receive the herpes zoster (HZ) vaccine in online settings. 0 (white rounded rectangle), not willing to receive the HZ vaccine; 1 (green rounded rectangle), willing to receive the HZ vaccine; HZ, herpes zoster.

**Figure 3:**
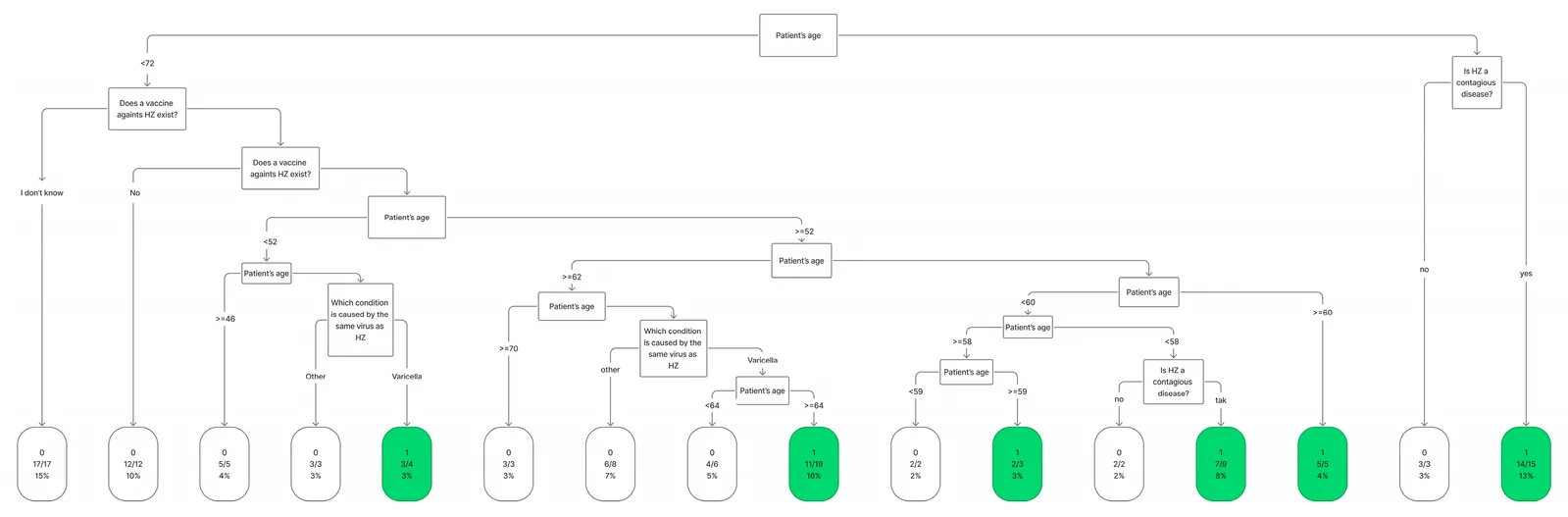
CART model predicting willingness to receive the herpes zoster (HZ) vaccine in onsite settings. 0 (white rounded rectangle), not willing to receive the HZ vaccine; 1 (green rounded rectangle), willing to receive the HZ vaccine; HZ, herpes zoster.

The classification tree shows how responses on HZ awareness, age, and comorbidities predict willingness to receive RZV with high specificity and sensitivity in both online (Figure 2; Table 9) and onsite settings (Figure 3; Table 9). For the online dataset, the selected tree parameters were: cp = 0.022, xerror = 0.525, and xsd = 0.083. For the outpatient clinic tree: cp = 0.028, xerror = 0.851, and xsd = 0.109.

## 4. Discussion

Considering potential differences in population characteristics between online and onsite groups, as described in the literature [56,57,58,59,60,61], we aimed to assess population discrepancies between these groups and to evaluate whether such differences are correlated with levels of knowledge regarding HZ and willingness to vaccinate.

A history of HZ was significantly associated with knowledge in at least half of the assessed items in both groups. Symptom severity and duration, as well as selected chronic diseases, were also significantly associated with knowledge in several items in both groups. Participants who had previously been vaccinated or were willing to be vaccinated more frequently provided correct responses to HZ- and vaccination-related knowledge items in both groups. These findings suggest that personal experience with HZ and selected clinical characteristics may be associated with greater awareness of the disease and its prevention.

Our findings are consistent with previous research indicating that, although general awareness of HZ is relatively high, detailed knowledge—particularly regarding vaccination—remains insufficient [50,51]. Across diverse settings, studies have shown that willingness to vaccinate is positively associated with physician recommendation, perceived disease severity, and prior experience with HZ, while major barriers include lack of awareness, safety concerns, and insufficient information [37,39,41,42,50,52,53,62,63,64,65,66,67,68]. In our study, the most frequently reported reasons for not receiving HZ vaccination were lack of awareness that a vaccine exists, concerns about vaccine side effects, and not receiving information from a physician or other medical staff. These findings highlight several areas that warrant greater attention in efforts to improve HZ vaccination uptake. Patients should receive clear information not only about the availability of vaccination, but also about its expected benefits, safety profile, and the clinical factors that increase the risk of HZ. The relatively frequent indication that no information had been provided by a physician or other healthcare professional suggests that opportunities for discussing HZ prevention may still be missed in routine practice. Such discussions should therefore include an assessment of vaccination status and eligibility, together with careful attention to patients’ concerns, particularly those related to adverse effects and the perceived value of vaccination in individuals at increased risk of HZ. Moreover, educational programs should incorporate these elements for both the general population and healthcare professionals involved in vaccination counselling. Some studies found no clear connection between prior VZV infection and vaccination decisions [54,69], possibly due to the mistaken belief that one infection provides lifelong immunity [37]. Notably, patients with chronic conditions, despite being at increased risk [70,71,72,73,74,75,76,77,78], often demonstrate limited awareness of their susceptibility and of available preventive measures [79,80]. However, some studies show that comorbidities could be associated with willingness to vaccinate [65,81]. Together, these observations indicate that both disease experience and access to reliable information may be relevant to vaccination attitudes. Educational efforts may be particularly relevant for individuals who have not previously experienced HZ, patients with chronic conditions who may not recognize themselves as being at increased risk, and individuals who have had limited opportunities to discuss HZ prevention with healthcare professionals.

Given the role of healthcare professionals in vaccination decision-making, we also examined changes in vaccination intention following onsite medical consultation. One of the most important findings of this study is the observed change in vaccination intention following medical consultation. Over 40% of initially hesitant or unwilling participants declared a positive change in their decision following consultation, while only a minority adopted a more negative stance. This underscores the critical role of healthcare professionals in addressing vaccine hesitancy and suggests that vaccination intentions may change following brief, targeted educational interventions. Importantly, 17.4% of participants reported a negative change in vaccination intention following consultation. The reasons for this change were not assessed in the present study, but the finding suggests that both the content and manner of counselling deserve further attention. Greater emphasis on individual HZ risk, balanced discussion of vaccine benefits and adverse effects, and careful exploration of patients’ specific concerns may help improve the response to counselling. Allowing sufficient time for questions and shared decision-making may also be important, particularly in patients who remain uncertain about vaccination. Given the observational nature of the study and the use of vaccination intention as the assessed outcome, these findings should be interpreted as associations rather than causal effects. They are nevertheless consistent with prior studies emphasizing the importance of physician recommendation as a key driver of vaccine uptake. Future studies should examine which elements of counselling are most effective and whether they translate into actual vaccine uptake.

Despite substantial demographic and clinical differences between the online and onsite groups, several patterns of association with HZ knowledge and vaccination attitudes were qualitatively similar across recruitment settings. A notable difference concerned the source of information, with online participants significantly more likely to rely on the internet. These differences should be considered when developing educational strategies for different patient groups.

An additional strength of this study is the application of CART analysis, which allowed for the identification of simple and clinically interpretable combinations of predictors associated with vaccination willingness. The good discriminative performance of the models suggests that a limited number of variables—such as awareness and basic health characteristics—may be sufficient to identify individuals who would benefit most from targeted education and counselling.

Overall, these findings highlight the need for improved educational strategies addressing gaps in knowledge about HZ and its prevention. In particular, increasing physician engagement and optimizing communication through both traditional and digital channels may play a key role in improving vaccination willingness.

### Limitations

Despite considerable effort devoted to preparing this study, several limitations must be acknowledged. First, the numbers of patients with individual chronic diseases were heterogeneous, and the number of participants with less common conditions was limited, reflecting their prevalence in the general population and restricted access to such patients.

In addition, most analyses were cross-sectional, which limits the ability to establish temporal relationships between the assessed factors and vaccination-related outcomes. Accordingly, the observed associations should not be interpreted as causal.

Second, the aim was to assess how different settings relate to knowledge and attitudes towards vaccination, accounting for baseline differences between groups, including age and place of residence. Although the observed age differences were expected, as online participants tend to be younger than primary care patients, selection bias may still be present. Similarly, differences in place of residence were anticipated, as online participation was not geographically restricted, whereas most patients in the Medical Center Medyk network reside in urban areas. The online group was recruited using non-probability, self-selected participation, which may further limit representativeness. Residual confounding by unmeasured or incompletely controlled demographic and clinical factors cannot be excluded. Consequently, the generalizability of the findings beyond the sampled Polish populations may be limited.

Moreover, individuals who participate in online surveys may be more health-conscious or motivated (volunteer bias) and may have higher digital literacy (digital literacy bias). Several clinical variables, including HZ history, comorbidities, and vaccination status, were based on participant-reported information and may therefore be subject to reporting error and recall bias. Despite technical safeguards, including limiting responses to one per IP address and applying logical consistency checks, multiple submissions (e.g., via VPNs or shared networks) and inattentive responses cannot be fully excluded. Questionnaires with inconsistent validation responses were excluded from analysis. However, although internal consistency checks were incorporated into the survey, the questionnaire was not subjected to formal psychometric validation. The large number of statistical comparisons should also be considered when interpreting the results, as multiple testing may increase the likelihood of statistically significant findings occurring by chance.

The consultation component has additional limitations. It did not include a non-consultation comparison group, and the assessed outcome reflected stated vaccination intention rather than subsequent vaccine uptake. Therefore, changes observed following consultation should not be interpreted as evidence of a causal effect of the consultation.

Finally, although CART model performance was assessed using cross-validation and bootstrap validation, some degree of overfitting cannot be excluded, and the models were not externally validated in an independent cohort. Future studies are therefore needed to confirm these findings.

## 5. Conclusions

Awareness of VZV and personal experience with HZ influence the likelihood of accepting vaccination. Previous studies have reported associations between HZ- and vaccine-related knowledge and willingness to be vaccinated [34,80]. Research indicates that individuals with better education are more likely to express intent to vaccinate [80,82,83,84]. Understanding of these aspects highlights the importance of education provided by healthcare professionals [55,63,85,86].

Over 40% of initially hesitant patients demonstrated a positive change in vaccination intention following medical consultation, reinforcing the pivotal role of the physician–patient interaction in HZ prevention. CART models identified vaccination awareness and age as the primary determinants of vaccination willingness, achieving excellent discriminative performance in both settings (AUC 0.84–0.86). Targeted, physician-delivered educational interventions—supplemented by digital communication for younger, online-active populations—represent the most promising pathway to improving HZ vaccination willingness in Poland and comparable settings.

## Figures and Tables

**Table 1:** Odds ratios (OR; 95% CI) and *p*-values for correct responses to HZ knowledge items by HZ history status, in onsite and online settings.

Setting	Question	Q No.	Yates-Corrected χ^2^ *p*-Value	OR for Correct Response	95% CI Lower for OR	95% CI Upper for OR	*p*-Value for OR
Onsite	Is HZ a contagious disease?	15	<0.0001 *	6.235	3.196	12.164	<0.0001 *
	Which disease is caused by the same virus as HZ?	16	0.0001 *	2.848	1.620	5.007	0.0003 *
	Are people aged ≥50 years at higher risk of HZ?	17	0.023	3.067	1.338	7.028	0.008 *
	Does a vaccine against HZ exist?	19	<0.0001 *	5.377	1.670	17.319	0.004 *
	What is the purpose of HZ vaccination?	21	0.014 *	4.200	1.495	11.797	0.006 *
Online	Is HZ a contagious disease?	15	0.004 *	2.526	1.370	4.660	0.003 *
	Which disease is caused by the same virus as HZ?	16	0.286	-	-	-	NS
	Are people aged ≥50 years at higher risk of HZ?	17	0.028	1.901	1.130	3.198	0.015 *
	Does a vaccine against HZ exist?	19	0.012 *	1.919	1.052	3.499	0.033
	What is the purpose of HZ vaccination?	21	0.429	-	-	-	NS

* Significant difference; NS, not statistically significant; HZ, herpes zoster. Only questions showing statistical significance in at least one setting are reported.

**Table 2:** Bivariable analyses of herpes zoster severity, symptom duration, and complications in relation to responses to knowledge questions in online and onsite settings.

	Question	Q No.	Onsite χ^2^	Onsite *p*-Value	Online χ^2^	Online *p*-Value
HZ severity	Is HZ a contagious disease?	15	7.16	0.023 *	2.29	0.33
	Which disease is caused by the same virus as HZ?	17	2.43	0.297	6.57	0.037 *
	Are people aged ≥50 years at higher risk of HZ?	19	5.43	0.067	14.80	0.0006 *
HZ symptom duration	Are people aged ≥50 years at higher risk of HZ?	17	10.674	0.3835	20.927	0.0216 *
	Does a vaccine against HZ exist?	19	15.743	0.1072	31.37	0.0005 *
	What is the purpose of HZ vaccination?	21	47.661	<0.0001 *	10.243	0.4195
HZ complications	Which disease is caused by the same virus as HZ?	16	69.756	<0.0001 *	10.607	0.9988
	Are individuals with chronic diseases at higher risk of HZ?	18	51.207	<0.0001 *	15.557	0.3411
	Does a vaccine against HZ exist?	19	59.522	<0.0001 *	11.43	0.652
	What is the purpose of HZ vaccination?	21	52.518	<0.0001 *	20.08	0.1277

* Significant difference.

**Table 3:** Bivariable analysis of individual chronic diseases and responses to selected knowledge questions.

Knowledge Question	Chronic Disease	Onsite *p*-Value	Online *p*-Value
Which disease is caused by the same virus as HZ?	Hypertension	0.013 *	0.059
	Autoimmune thyroid disease	0.005 *	0.203
Are people aged ≥50 years at higher risk of HZ?	Type 2 diabetes	0.213	0.016 *
Are individuals with chronic diseases at higher risk?	Chronic lung disease	0.088	0.028 *
	Type 2 diabetes	0.372	0.009 *
Does a vaccine against HZ exist?	Hypertension	0.205	0.0001 *
	Type 2 diabetes	0.010 *	0.051

* Significant difference. Prior vaccination status and willingness to receive HZ vaccination differed significantly across responses to several disease- and vaccine-related knowledge items in both groups (Supplementary Table S2).

**Table 4:** Changes in willingness to vaccinate against herpes zoster after onsite medical consultation (*n* = 184).

Outcome	*n* (%)
Persons who did not change their opinion	57 (30.98)
Persons willing to vaccinate regardless of physician’s opinion	16 (8.70)
Persons who changed their opinion positively	79 (42.93)
Persons who changed their opinion negatively	32 (17.40)

**Table 5:** (**a**) Effect of onsite medical consultation on herpes zoster vaccination willingness by clinical characteristics; (**b**) Effect of onsite medical consultation on herpes zoster vaccination willingness by pain features.

**(a)**					
**Characteristic**	**Did Not Change *n* (%)**	**Willing Regardless *n* (%)**	**Changed Positively *n* (%)**	**Changed Negatively *n* (%)**	***p*** **-Value**
Hypertension	18 (31.58)	11 (68.75)	33 (41.77)	20 (62.50)	0.005 *
Any comorbidity	40 (70.18)	16 (100)	69 (87.34)	28 (87.50)	0.002 *
History of HZ	20 (35.09)	16 (100)	43 (54.43)	15 (46.88)	<0.0001 *
**(b)**					
**Pain Duration**	**Did Not Change *n* (%)**	**Willing Regardless *n* (%)**	**Changed Positively *n* (%)**	**Changed Negatively *n* (%)**	***p*** **-Value**
<7 days	1 (5.0)	0 (0)	1 (6.67)	-	0.0005 *
7 days–2 weeks	4 (20.0)	0 (0)	6 (13.95)	2 (13.33)	
2 weeks–1 month	12 (60.0)	7 (43.75)	18 (41.86)	9 (60.0)	
1–3 months	3 (15.0)	3 (18.75)	16 (37.21)	3 (20.0)	
>3 months	0 (0)	6 (37.50)	3 (6.98)	0 (0)	
Pain intensity median (IQR)	6 (5–8)	8 (7–8)	8 (7–9)	7 (6–8)	0.012 *

* Significant difference. IQR, interquartile range.

**Table 6:** Sources of information about the herpes zoster vaccine in onsite and online settings.

Source of Information	Onsite *n* (%)	Online *n* (%)	*p*-Value
From a physician or healthcare professional	98 (46.89)	92 (39.48)	0.0099 *
From friends or family	13 (6.22)	21 (9.01)	
From the internet or other media	33 (15.79)	66 (28.33)	
I was not aware that a vaccine existed	63 (30.14)	52 (22.32)	
From another source	2 (0.96)	2 (0.86)	

* Overall *p*-value for group comparison.

**Table 7:** Main reasons for not receiving the herpes zoster vaccine among unvaccinated participants in onsite and online settings.

Reason	Onsite *n* (%)	Online *n* (%)
I was not aware that a vaccine existed	64 (35.15)	59 (32.24)
The cost of vaccination	6 (3.30)	6 (3.28)
I did not receive information from a doctor/medical staff	39 (21.43)	38 (20.77)
I did not know I was in a risk group	15 (8.24)	7 (3.83)
Concern about vaccine side effects	45 (24.73)	48 (26.23)
I am not worried about HZ course or complications	9 (4.95)	19 (10.38)
Concern about insufficient vaccine effectiveness	4 (2.20)	6 (3.28)

**Table 8:** Main reasons for receiving the herpes zoster vaccine among vaccinated participants in onsite and online settings.

Reason	Onsite *n* (%)	Online *n* (%)
Previous episode of HZ	7 (26.92)	12 (26.67)
Fear of developing HZ and its complications	8 (30.77)	16 (35.56)
Recommendation from a physician or healthcare professional	8 (30.77)	16 (35.56)
Recommendation from family or friends	1 (3.85)	1 (2.22)
Illness of a relative or friend	2 (7.69)	0 (0)
Reimbursement	0 (0)	0 (0)
Information obtained from the Internet	0 (0)	0 (0)

**Table 9:** Performance metrics of the CART model predicting willingness to receive the herpes zoster (HZ) vaccine in online and onsite settings.

Metric	Online Group, Value (95% CI)	Onsite Group, Value (95% CI)
	Value (95% CI)	Value (95% CI)
AUC	0.8415 (0.7772–0.9035)	0.8611 (0.8076–0.9022)
Accuracy	0.8462 (0.7678–0.9062)	0.8561 (0.7844–0.9111)
Sensitivity	0.8936 (0.8106–0.9316)	0.9178 (0.7898–0.9928)
Specificity	0.8143 (0.6843–0.9183)	0.7797 (0.6687–0.8627)
Positive predictive value	0.7636 (0.7167–0.8415)	0.8375 (0.7815–0.9390)
Negative predictive value	0.9194 (0.9139–0.9224)	0.8906 (0.8827–0.8958)
Overall error	0.1538	0.1439

## Data Availability

Data are contained within the article or Supplementary Material.

## Supplementary Materials

The following supporting information can be downloaded at: https://www.mdpi.com/article/10.3390/vaccines14090834/s1, Supplementary Table S1: Demographic and clinical characteristics of onsite and online respondents.; Supplementary Table S2: Bivariable analysis of prior HZ vaccination, vaccination willingness, and responses to knowledge items in both study groups.

## Abbreviations

The following abbreviations are used in this manuscript:

ACIP	Advisory Committee on Immunization Practices
CART	Classification and Regression Tree
HZ	Herpes Zoster
HZO	Herpes Zoster Ophthalmicus
RZV	Recombinant Zoster Vaccine
ZVL	Zoster Vaccine Live
