# Supplementary material for: Knowledge of Herpes Zoster and Attitudes Toward Vaccination: A Questionnaire-Based Study in Outpatient and Online Settings
Source: Vaccines (Basel). 2026 Sep 21;14(9):834. doi: 10.3390/vaccines14090834 (PMC13612019; doi:10.3390/vaccines14090834)
Supplement: Supplementary file 1 [file vaccines-14-00834-s001.zip › Supplementary Table S1.pdf]

Supplementary Table S1. Demographic and clinical characteristics of onsite and online respondents.

Variable n(%)	Onsite (n=212)	Online (n=241)	p-value
Basic characteristics			
Men	68 (32.08)	97 (40.25)	0.0713
Women	144 (67.92)	144 (59.75)	
Respondents living in			
Urban area	147 (69.34)	120 (49.79)	<0.0001 *
Suburban area	42 (19.81)	65 (26.97)	
Rural area	23 (10.85)	56 (23.24)	
Age group			
18–49 years	36 (16.98)	112 (46.47)	<0.0001 *
50–59 years	51 (24.06)	77 (31.95)	
60–69 years	82 (38.68)	40 (16.60)	
70–79 years	31 (14.62)	10 (4.15)	
≥80 years	12 (5.66)	2 (0.83)	
Chronic diseases **			
Haematologic/lymphatic malignancy	4 (1.89)	8 (3.32)	0.3434
Solid organ transplant recipient	1 (0.47)	4 (1.66)	0.2272
HSCT recipient	1 (0.47)	4 (1.66)	0.2272
Solid organ malignancy	8 (3.77)	10 (4.15)	0.8381
Hypertension	97 (45.75)	80 (33.22)	0.0063 *
Chronic heart disease	26 (12.26)	30 (12.45)	0.9527
Chronic liver disease	5 (2.36)	6 (2.49)	0.9279
Chronic lung disease	17 (8.02)	10 (4.15)	0.0826
Chronic kidney disease	8 (3.77)	17 (7.05)	0.1271
Type 1 diabetes	3 (1.42)	13 (5.39)	0.0221 *
Type 2 diabetes	58 (27.36)	33 (13.69)	<0.0001 *
Depression	15 (7.08)	8 (3.32)	0.0692
Autoimmune thyroiditis	16 (7.55)	20 (8.30)	0.7679
Rheumatoid arthritis	5 (2.36)	11 (4.56)	0.2044
Ulcerative colitis or Crohn's disease	1 (0.47)	3 (1.24)	0.3801
Systemic lupus erythematosus	5 (2.36)	19 (7.88)	0.009 *
Ankylosing spondylitis	4 (1.89)	6 (2.49)	0.663
Other autoimmune disease or other immunodeficiency	2 (0.94)	0 (0)	0.33
None of the above	37 (17.45)	43 (17.84)	0.9136
Immunosuppressive medications **			
csDMARDs only	4 (1.89)	18 (7.47)	0.008 *
bDMARD only	2 (0.94)	2 (0.83)	0.8551
bDMARD + csDMARD	2 (0.94)	7 (2.90)	0.1569
Oral corticosteroids	14 (6.60)	28 (11.62)	0.0967
Other immunomodulatory drugs	7 (3.30)	14 (5.81)	0.2522
None of the above	164 (77.36)	157 (65.56)	<0.0001 *

Patient unsure	13 (6.13)	22 (9.13)	0.2147
History of herpes zoster			
Yes	104 (49.06)	102 (42.32)	0.1673
No	105 (49.53)	131 (54.36)	
Patient unsure	3 (1.42)	8 (3.32)	
HZ vaccination status			
Unvaccinated	184 (88.04)	188 (80.69)	0.0331*
Vaccinated	25 (11.96)	45 (19.31)	

* Significant difference; ** More than one answer could be selected; HSCT, haematopoietic stem cell transplantation; csDMARDs, conventional synthetic disease-modifying antirheumatic drugs; bDMARD, biological disease-modifying antirheumatic drug.
