# Supplementary material for: Knowledge of Herpes Zoster and Attitudes Toward Vaccination: A Questionnaire-Based Study in Outpatient and Online Settings
Source: Vaccines (Basel). 2026 Sep 21;14(9):834. doi: 10.3390/vaccines14090834 (PMC13612019; doi:10.3390/vaccines14090834)
Supplement: Supplementary file 1 [file vaccines-14-00834-s001.zip › Supplementary Table S2.pdf]

Supplementary Table S2. Bivariable analysis of prior HZ vaccination, vaccination willingness, and responses to knowledge items in both study groups.

Setting	Question	Q No.	Prior vacc: χ^2	Prior vacc: p	Willingness: χ^2	Willingness: p
Onsite	Is HZ a contagious disease?	15	0.3	0.874	12.7	0.013 *
	Which disease is caused by the same virus as HZ?	16	7.3	0.120	15.7	0.046 *
	Are people aged ≥50 years at higher risk of HZ?	17	5.0	0.080	1.3	0.868
	Does a vaccine against HZ exist?	19	12.3	0.002 *	16.3	0.003 *
	What is the purpose of HZ vaccination?	21	4.2	0.122	7.988	0.092
Online	Is HZ a contagious disease?	15	6.7	0.035 *	7.4	0.117
	Which disease is caused by the same virus as HZ?	16	4.5	0.343	5.4	0.711
	Are people aged ≥50 years at higher risk of HZ?	17	7.7	0.021 *	11.3	0.023 *
	Does a vaccine against HZ exist?	19	22.0	<0.0001 *	22.6	0.0001 *
	What is the purpose of HZ vaccination?	21	7.0	0.030 *	10.0	0.040 *
